# The Machine Speaks: Conversational AI and the Importance of Effort to Relationships of Meaning

**DOI:** 10.2196/53203

**Published:** 2024-06-18

**Authors:** Anna Hartford, Dan J Stein

**Affiliations:** 1 Neuroscience Institute University of Cape Town Cape Town South Africa; 2 South African Medical Research Council Unit on Risk and Resilience in Mental Disorders Department of Psychiatry University of Cape Town Cape Town South Africa

**Keywords:** artificial intelligence, AI, conversational AIs, generative AI, intimacy, human-machine interaction, interpersonal relationships, effort, psychotherapy, conversation

## Abstract

The focus of debates about conversational artificial intelligence (CAI) has largely been on social and ethical concerns that arise when we speak to machines—what is gained and what is lost when we replace our human interlocutors, including our human therapists, with AI. In this viewpoint, we focus instead on a distinct and growing phenomenon: letting machines speak for us. What is at stake when we replace our own efforts at interpersonal engagement with CAI? The purpose of these technologies is, in part, to remove effort, but effort has enormous value, and in some cases, even intrinsic value. This is true in many realms, but especially in interpersonal relationships. To make an effort for someone, irrespective of what that effort amounts to, often conveys value and meaning in itself. We elaborate on the meaning, worth, and significance that may be lost when we relinquish effort in our interpersonal engagements as well as on the opportunities for self-understanding and growth that we may forsake.

## Introduction

Conversation is central to our shared humanity. It is the means through which we make ourselves knowable to another and come to know them in turn. Our mental states—our beliefs, feelings, intentions, desires, and attitudes—are in some respects unreachable by another and sometimes even opaque to ourselves. However, in conversation, we render them articulable, and therefore, accessible. Not unrelatedly, in these exchanges, we often learn about ourselves as well as the other person. The recent emergence of powerful conversational artificial intelligences (CAIs) has therefore been unsettling on various levels (far more so than equally powerful AIs that operate in mediums besides conversation). In their extraordinary replication of the means through which we express our mental states, it is tempting to impute these states to our AI interlocutors. After all, the articulation of thinking (or feeling, hoping, willing, and desiring) is usually all the evidence we require to attribute the relevant mental states to someone.

In her book, Reclaiming Conversation: The Power of Talk in a Digital Age, Sherry Turkle [1] endeavors to make the case for conversation in a world that has increasingly abandoned it for the conveniences (and safeties) of mere digital connection. “At a first, we speak through machines and forget how essential face-to-face conversation is to our relationships, our creativity, and our capacity for empathy,” Turkle writes. “At a second, we take a further step and speak not just through machines but to machines. This is the turning point” [1]. This concern was prescient, and Turkle has more recently elaborated on it with reference to the proliferation of CAIs or social chatbots, such as Xiaoice, Woebot, or Replika. These CAIs aim to provide intimacy, but of what sort? Turkle suggests that this intimacy is necessarily fraudulent since it is (by design) devoid of the emotional vulnerability crucial to genuine intimacy [2]. Similarly, these CAIs eliminate the demands and challenges of empathy required for genuine interpersonal exchanges [1,3]. These arguments align with Turkle’s long-standing critique of how computers affect our relationships with ourselves and with others [3-5].

## “Speaking to Machines”: CAIs and the Possibility of Insight

There is ongoing debate concerning the type and quality of conversations possible with CAIs and their appropriateness in therapeutic contexts. In psychotherapy, the digitization of many processes may suggest that CAIs can simply replace the therapist. However, it is also possible to argue that the psychotherapeutic relationship and the experience of that relationship are what is most crucial. In psychodynamic psychotherapy, the client experiences transference while the therapist experiences counter-transference, and working through these processes leads to therapeutic change. In frameworks more influenced by cognitive-behavioral principles, such as schema therapy, the therapist may play a key role in providing “reparenting,” a process that leads to positive outcomes.

Ethical concerns with CAIs in therapeutic contexts include the biases and other harmful prompts that might arise in such exchanges, along with the potential dearth of responsibility and accountability for these harms [6-10]. However, even if such patent ethical concerns were addressed or eradicated, central questions would persist: what sort of presence or entity do we have in CAIs? [11-14] and perhaps relatedly, is it possible for CAIs to facilitate genuine self-knowledge, self-understanding, and insight in their human interlocutors?

Some have suggested that engagements with CAIs are necessarily deficient in this crucial respect, especially if we consider the practice of joint attention, as well as other forms of mutual recognition and acknowledgment, to be central to the therapeutic conversation (and indeed to conversation more generally) [11,14-16]. Relatedly, there are concerns about the lost mutuality of these exchanges [16,17]. Conversations with bots do not demand that we empathize with or accommodate another, since, in an important sense, there is no one else there [14,16,18]. As Andrew McStay [18] points out, much depends on the account of empathy we are assuming. McStay argues that accounts that are more accommodative of CAIs are “deficient and potentially dangerous” insofar as they lack interdependence, copresence, and particularly moral responsibility [18].

However, others disagree with these characterizations and see no reason why CAIs cannot encourage genuine introspection [19-21]. What is required in the therapeutic exchange—according to some of these proponents—is not necessarily mutual agency, but rather the experience of being emotionally supported and encouraged to engage in self-reflection [20,21]. To necessitate another subjectivity, or the presence of another full-fledged agent, is to presuppose the illegitimacy of CAIs in these contexts, and to needlessly curtail the possibilities of what qualifies as a genuine therapeutic conversation. After all, human therapists regularly fail to generate the conditions for self-understanding and insight, irrespective of their full-fledged agency [19].

We find these counterarguments compelling. Furthermore, if therapeutic benefits are possible through CAIs—as some research suggests [22-24] (although far more investigation is required [25])—then we potentially have a powerful tool in therapeutic CAIs. Given the immense shortfall in mental health care globally [26,27] and the often prohibitive cost of undertaking conventional psychotherapy, we would be remiss to hastily disregard the beneficial possibilities of therapeutic CAIs. Moreover, certain individuals and populations might experience unique benefits from the format of engagement required by therapeutic exchanges with CAIs, and (relatedly) may not experience the particular advantages of in-person conversation highlighted by advocates such as Turkle (this point has been made with regard to children on the autism spectrum in particular [28,29]).

## Being Spoken for: CAIs and Surrendering Articulation

In recent reckonings with the rise of CAIs, the focus has generally been on concerns like those outlined above: what becomes of us when we increasingly replace our human interlocutors—including our human therapists—with AIs, that is (in Turkle’s phrase) “when we speak not just through machines but to machines” [1].

Our central concern in this viewpoint, however, is different. Although certain dimensions of the preceding debate are of relevance to our position, we can also remain agnostic with regard to the value of “speaking to machines,” whether in a therapeutic context or otherwise. We can remain open to the possibility that bot and human engagements can generate genuine depth, worth, and meaning. Furthermore, we need not presume that the conditions for the emergence of genuine self-understanding and self-reflection cannot be generated in interactions with CAIs. Rather, our concern arises independently, for we now seem to have reached another turning point, and one that extends even further. “At a third point,” we might add to Turkle’s list, “we take yet another step and let machines speak for us” [1].

We will concentrate on the significance of these forces to our self-knowledge and our interpersonal relationships, although more could be, and has been, said about their implications more generally, for example, concerning achievement gaps (where automation threatens to undermine genuine achievement, and therefore, meaningful work [30]) and responsibility gaps (where automation threatens to undermine responsibility for harmful outcomes [31,32]).

Our central concern will be the following: what is potentially at stake, personally and interpersonally, when we let the machine speak for us? We will explore this question within the framework of philosophical and ethical debates concerning the interpersonal value of effort, rather than exploring it qualitatively or quantitatively (although further empirical research on these questions would be valuable).

This third transition can take many forms, some seemingly more trivial than others. When we are writing an email and the remainder of the sentence auto-fills in gray, we are tempted to stop speaking for ourselves and let the machine speak for us instead.

At times, the costs of this surrender may seem slight, if they exist at all. What does it matter if you articulate some rote phrase to a distant work acquaintance or have it articulated for you instead? However, in other circumstances and other relationships, even these subtle interventions can carry weight.

In an early exploration of the implications of large language models—written in 2019, before the mass rollout of ChatGPT and other large language models—the journalist John Seabrook [33] wrote the following about the experience of using Smart Compose to autocomplete his emails:

Finally, I crossed my Rubicon. The sentence itself was a pedestrian affair. Typing an e-mail to my son, I began “I am p—” and was about to write “pleased” when predictive text suggested “proud of you.” I am proud of you. Wow, I don’t say that enough. And clearly Smart Compose thinks that’s what most fathers in my state say to their sons in e-mails. I hit Tab. No biggie.

And yet, sitting there at the keyboard, I could feel the uncanny valley prickling my neck.

Nowadays, the modes in which the machine can speak for us have expanded enormously from these first modest iterations. There are many examples to consider, and many more are developing as we write, but the ways through which we can outsource the labor of our interpersonal articulations are currently expanding exponentially.

Take one example: it is now possible to get CAIs to message on your behalf on dating apps. A variety of start-ups have generated different tools that allow you to hand over your messages to an AI [34]. Instead of having to initiate a conversation with a prospective date—or to come up with thoughtful or witty replies to their messages—AI will do it for you.

When you do not care for the people you are messaging, this option offers a certain pragmatic appeal (especially given the volume of messaging that contemporary dating apps necessitate). However, when you *do* care about a person, the temptation might be even stronger. The CAI, after all, always has an idea of what to say next, and moreover, it offers a version of what you *should* say—a statistically probable representation of what *people like you* say at *times like this*. In comparison, speaking for yourself can feel risky. The things you might say on your own—the way in which you try to make yourself known and get to know others—might be odd, off-putting, or *wrong* somehow.

Take another example: in June 2023, *The New York Times* reported that some doctors were turning to AI to communicate compassionately with patients [35]. We have all experienced the sense of inadequacy that comes with trying to say something supportive to someone who is in an awful circumstance. At such times, we can cast around for ages and summon nothing but cliches. How alluring it is to have a ready-made response instead, and one so well trained in the performance of genuine feeling. The AI’s messages will be, in many cases, much better than what we could have produced on our own—kinder, more thoughtful, and more encouraging. Yet no matter how superbly it manages to express care and compassion, this expression is of course divorced from any genuine experience of care and compassion. We should be cautious, in our expedient outsourcing of this emotional connection and engagement, of when we begin to divorce ourselves from the genuine experience of care and compassion along with it.

When we are struggling to find the right thing to say, it may feel like we are achieving nothing. Yet it is precisely in these times—as we try to understand what someone else is enduring, to feel for them, and to express that feeling—that we are undertaking the genuine experience of care and compassion, without which the words themselves are hollow.

One optimistic response is that we might learn more empathetic engagement from the example of the machines. However, this seems unlikely. It is like suggesting that we will improve our spelling skills by relying on automated spell-check or that we will remember more phone numbers through the excellent example set by our phones. Of course, we will not, as the process removes effort, and little of importance has ever been learned without effort.

Thinking ahead—and not necessarily too far ahead—it is possible to see how the temptation to let the machine speak might overspill our text-based conversations. The push to normalize mixed-reality engagements—most notably with the launch of Apple’s Vision Pro headset last year—would make it possible for the machine to take over not only our text-based correspondence but also our face-to-face conversations.

We are, right now, at the initial stages of the temptation to begin ceding our expressions to CAIs. However, with little imagination, it is easy to see all the ways in which these temptations are poised to grow. After all, if it was largely the machine whose messages charmed someone into going on a date with you in the first place, how enticing would it be to let the machine keep on speaking when you have to go on the date yourself? The machine speaks with such authority, and as our confidence in its utterances grows, our confidence in our own could correspondingly diminish.

To our mind, the potential costs (to one’s own humanity and to our shared humanity) of CAIs are greatest when we allow them to speak for us. Genuine conversation nurtures authentic engagement with others and a better understanding of ourselves. Turkle [1] emphasizes what is lost when we speak through machines, and further still, when we speak *to* machines, but there is, even in these latter engagements, the possibility of coming to know our own thoughts and feelings, of having to search for, and to find, the expression for our experience, and recognizing that the experience precedes the expression that follows.

However, when we allow the machine to speak for us, even this possibility diminishes. We can too easily avoid the effort it takes to genuinely understand ourselves and our unique circumstances (undertakings that are not necessarily discouraged by speaking *to* machines [20,21,29]). We are not encouraged to find the expression for our experience. Instead, we can too easily mistake whichever expressions we receive for our own experience, scarcely recognizing what we have lost in the exchange.

## Effort and Meaning

The purpose of these technologies is, in no small part, to remove effort. To take something that once required a great deal from us and make it require little to nothing. Effort is by definition a burden, and in any given instance of having to exert effort, we are always wishing there was a way to be rid of it, but effort also has enormous value, and in some cases, even intrinsic value. This can be true in many realms—there are crucial senses in which “achievement” itself is impossible without effort [30,36]—but it is especially true in our interpersonal relationships. In some interpretations, effort allows us to *reveal* our care and concern for one another and make it knowable. In such interpretations, its role is primarily epistemic. This epistemic role is not trivial in itself, but there are also interpretations whereby effort is more significant still—instead of only allowing us to *reveal* care and concern, it may also *generate* this care and concern [37,38]. Imagine a husband who lovingly cares for his wife through a long illness. His devotion through this ordeal might not only reveal the depths of his love for his wife, but it could also *generate* those depths.

In this sense, the exertion of effort might have both generative and revelatory value in our interpersonal relationships, and the relinquishment of effort might have serious costs on both fronts. To make an effort for someone, irrespective of what that effort amounts to, conveys value and meaning in itself. Many of our interpersonal practices are ways of trying to make real or manifest the effort that is in fact of genuine importance to us. In turn, when effort is removed from these practices, so is their worth.

Take one example: nowadays, Facebook provides automatic reminders of people’s birthdays. The moment this memory became automated, the fact of remembering someone’s birthday (which used to carry weight and significance) became increasingly meaningless. It is now possible to set up your account to automatically post a rote birthday message on the appropriate day; you need not even give the person a moment’s thought. These automated messages are equivalent, in terms of their interpersonal worth, to the automated birthday messages sent by a bank or a mobile service provider. Without requiring any thought or effort, the whole practice loses its significance. What other forms of interaction could we surrender to this fate, as we are increasingly able to opt for the effortless modes of expressing pride, love, affection, or consolation to the people around us?

## Conclusions

In turn, we should begin to think carefully (even if just for ourselves) about which of these technologies we choose to use, in different contexts and spheres of our lives, and which ones we do not. Where we choose to use them, we should think equally hard about the *manner* of our engagement and the extent of our agency within it; the more passive we allow ourselves to be, the greater the potential costs we have gestured to in this viewpoint. This is especially true when it comes to those undertakings that have value in and of themselves—rather than value only for their outputs [30]—and also, as we have emphasized in this viewpoint, when it comes to those relationships and human interactions in which our engaged presence, as well as our emotional and intellectual attention and reflection, carries so much significance.

There is an adage in developmental psychology: the toys that are best for children are the ones that require them to do the most work. “The best toys are 90% the kid, 10% the toy,” as psychologist Kathy Hirsh-Pasek put it. “If it’s 90% the toy, and 10% the kid, that’s a problem” [39]. The toys that demand the most of a child are the ones that generate creativity, teach them problem-solving, and encourage their social interactions. On the other hand, the toys that merely require a child to press a button will teach them only to press a button over and over again. When we consider children, we usually show special caution for what will aid and hamper their development, flourishing, and well-being. However, our development does not cease after childhood, and indeed, much of the hardest work (in learning to know as well as relate to ourselves and others) still lies ahead. Given that, we should perhaps pause and wonder what opportunities for self-development we might be forsaking as we embrace, ever more, the toys that want to do everything for us, while we ourselves do less and less. We should remember, in the ceaseless war against effort, that far from needing to be eradicated at every opportunity, there are spheres of our lives—and our interpersonal relationships are a prime example—where effort itself can be the whole point.

## Abbreviations

AI: artificial intelligence
CAI: conversational artificial intelligence
